# *Dacrycarpus* pattern shedding new light on the early floristic exchange between Asia and Australia

**DOI:** 10.1093/nsr/nwz060

**Published:** 2019-05-09

**Authors:** Xinkai Wu, Xiaoyan Liu, Tatiana Kodrul, Cheng Quan, Jianhua Jin

**Affiliations:** 1 State Key Laboratory of Biocontrol and Guangdong Key Laboratory of Plant Resources, School of Life Sciences, Sun Yat-sen University, China; 2 Geological Institute, Russian Academy of Sciences, Russia; 3 School of Earth Science and Resources, Chang’an University, China

The recent discovery of mummified *Dacrycarpus* remains from the Miocene Erzitang Formation of the Guiping Basin, Guangxi, southern China, essentially contests our concepts of floristic exchange between Australia and China. East Asia has several biodiversity hotpots and is a key region for understanding the origin and evolution of the biodiversity in the Northern Hemisphere [1]. Most Gondwanan elements in East Asia are those formerly distributed across much wide areas in the Southern Hemisphere, but their modern distributions are the results of complex geological and climatic changes. Understanding the biogeographical history of Gondwanan taxa, and the mechanism underpinning their current distributions, help in understanding and conserving extant biodiversity.

Nowadays, *Dacrycarpus* (Endlicher) de Laubenfels (Podocarpaceae) is trans-equatorially distributed in the western Pacific Region, from southernmost China to Fiji and New Zealand (Fig. 1) [2]. However, this genus is considered to be of Gondwanan origin, given that almost all the known megafossils have been documented from the Southern Hemisphere, especially from Australasia and South America (Fig. 1), with the earliest records from Eocene of Patagonia and Australia [3]. Such a remarkable distributional difference between the modern and geological range suggests that *Dacrycarpus* is a good example of cross-equatorial migration over extremely long distances and latitudinal shifts in Gondwanan biogeography. Recently, we discovered the first Northern Hemisphere *Dacrycarpus* megafossil record from the Miocene Erzitang Formation of Guiping Basin, Guangxi, low-latitude southern China. The remains are preserved as whole-plant, with mummified leafy shoots with dimorphic leaves, female cones and a male cone with *in situ* pollen (Fig. 2; taxonomic details will be published in a separate paper). The occurrence of these fossils solidly suggests the arrival of *Dacrycarpus* in Asia from the Southern Hemisphere at least by the Miocene, referred to as the *Dacrycarpus* pattern of paleophytogeography. By reviewing the biogeographic history of this genus, we here discuss the related geological and climate events and plant physiological characteristics underpinning this migration in deep time.

## PHYTOGEOGRAPHICAL HISTORY OF *DACRYCARPUS*

Due to the inherent limitations of the fossil record, previous studies concerning *Dacrycarpus* mainly focused on the origin of this genus and the internal transmission routes within Gondwana. Florin first proposed that both the fossil record and the modern distribution indicate that *Dacrycarpus* originated in the Southern Hemisphere [4]. The center of origin is largely located in a region between eastern Australia and New Zealand to western Antarctica, from where *Dacrycarpus* might have begun spreading northward along both sides of the South Pacific. Although *Dacrycarpus* became extinct in both South America and Antarctica around the Paleogene–Neogene transition and disappeared in eastern Australia in the Neogene, in the present day, it still survives on the islands to the east and north of Australia, with the highest diversity being in New Guinea [4]. Florin's hypothesis systematically expounded the origin and migration of *Dacrycarpus*. Moreover, plenty of Patagonian megafossils indicate that the origin center was confined to terminal Gondwana (Patagonia–Antarctica–Australia) [3].

**Figure 1.**
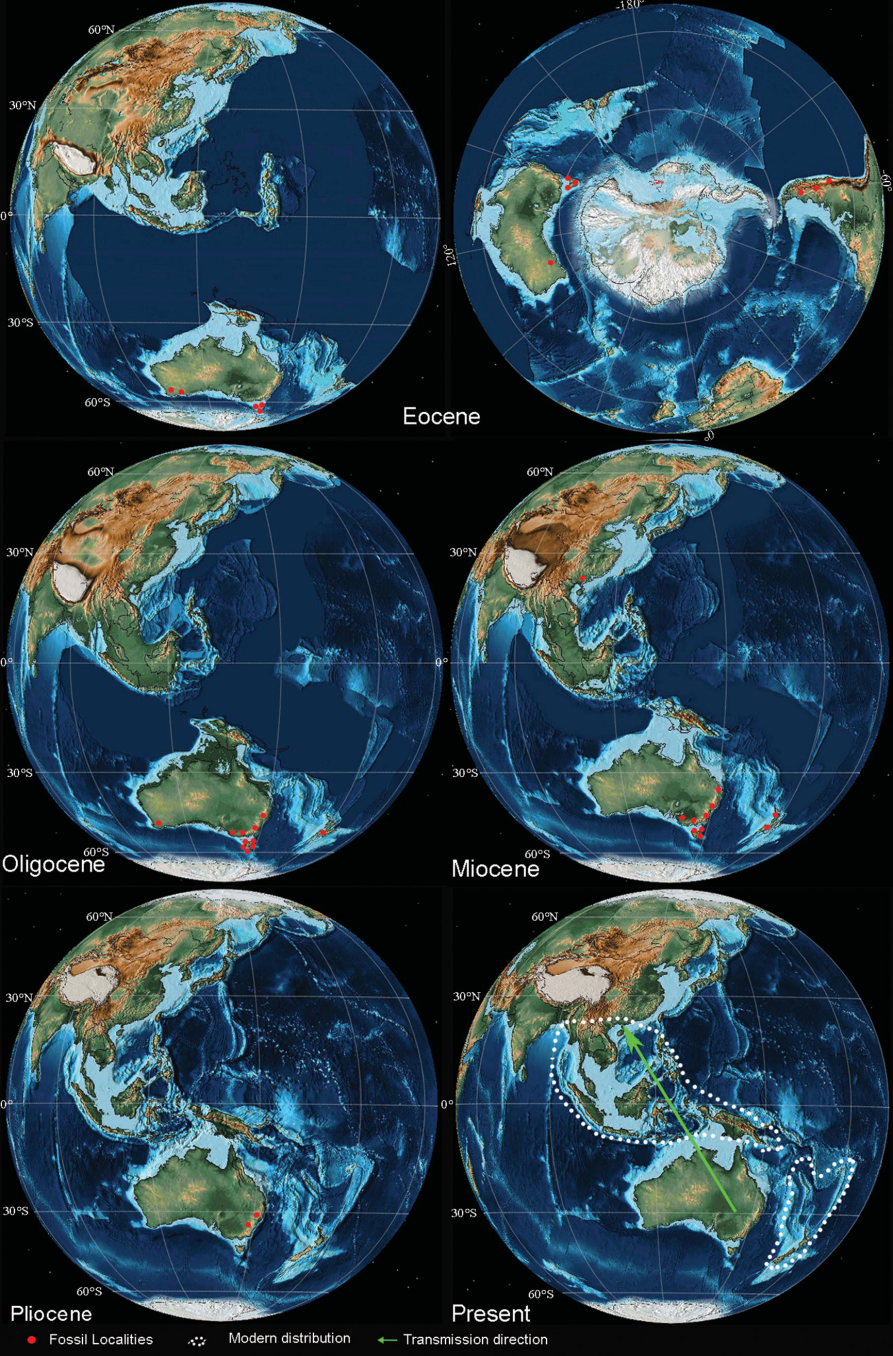
Paleogeographical maps of different periods showing the previous macrofossil records, the change of land bridge and the possible immigration route of *Dacrycarpus*. Data of paleomaps are available at EarthByte (https://www.earthbyte.org/).

Based on palynological data, Morley proposed that *Dacrycarpus* might have originated during the Late Cretaceous in Australasia and extended eastward to Patagonia during the early Eocene [5]. Along the west side of the Pacific Ocean, it had spread to New Guinea by the late Miocene and subsequently reached Borneo and Java in the early Pliocene. During the early Pleistocene, *Dacrycarpus* dispersed to Sumatra, the Malay Peninsula and Myanmar. However, the new fossil discovery from the Miocene of southern China indicates that *Dacrycarpus* has inhabited East Asia by the Miocene.

## RELATED GEOLOGICAL AND CLIMATIC EVENTS AS WELL AS PLANT PHYSIOLOGICAL CHARACTERISTICS

Geological events boost the physical pathways, allowing plant transmission. Contact between Australia and the Southeast Asian can be envisaged through the relationship between the Sunda and Sahul shelves. The Sunda Shelf has been an almost permanent landmass since the beginning of the Mesozoic and it formed a much larger emergent land area in the Late Cretaceous resulting from the addition of the continental fragments of Southwest Borneo and later East Java-West Sulawesi [6]. The accelerated northward movement of the Australian plate around 45 Ma resulted in subduction at the Sundaland margins, causing widespread land changes and rifting throughout Sundaland. During the Eocene to early Miocene, volcanic arcs existed at the southern margin of Sundaland, consisting mainly of islands rather than continuous and extensive areas of land from Sumatra to Sulawesi. Eastern Sundaland was separated from South China by the proto-South China Sea, resulting in the isolation of West Sulawesi from East Borneo. At about 25 Ma, the collision between the northern Australian margin, Sulawesi and the Halmahera Arc probably created a discontinuous land connection via island chains of the Halmahera Arc and the Philippines into Sulawesi. Together with the collision of the Melanesian arcs and the Ontong Java Plateau, Asia and Melanesia were also linked via a very long discontinuous island chain. The Miocene Sundaland–Australia collision in Sulawesi, and the subsequent collision with the margin of South China in North Borneo, led to the rearrangement or accretion of East Indonesia and the orogeny of Borneo, Sulawesi and the Banda Arc. This collision must have established the first links of Asia and Australia during the early Miocene, but there was no continuous land connection. Moreover, the consequential arrival of arcs from the Pacific made the islands of East Indonesia emerge. Subduction of the proto-South China Sea ended in the early Miocene and it led to a significant enlargement of the land area in Palawan and Borneo. Moreover, the subduction of the Celebes Sea beneath South Sabah and the Sulu arc gave rise to a mid- and late-Miocene intermittent volcanic island system that connected Borneo and the Philippines. For the majority of the Miocene, volcanoes in Sumatra and Java remained active and were separated from the mainland as islands. After greatly diminishing during the early to mid-Miocene, the Java-Sulawesi sector of the Sunda Arc resumed its volcanic activity at the end of the mid-Miocene and continued into the late Miocene. From about 15 Ma, the Java subduction zone began to roll back into the Banda embayment, causing a major extension in Sulawesi and fragmentation in the Sula spur. This might have resulted in abundant volcanic activity and renewed elevation on land. Southeast Sulawesi shows good evidence of emergence since the early Miocene, and the distribution and depths of water on the Sunda Shelf indicate that there were always routes from Borneo via Java to Sulawesi in the form of small islands [6]. A period of about 10 million years is probably the most likely time for connections to exist between Australia and Sulawesi, because of the relatively extensive areas of possible islands surrounded by shallow seas.

**Figure 2.**
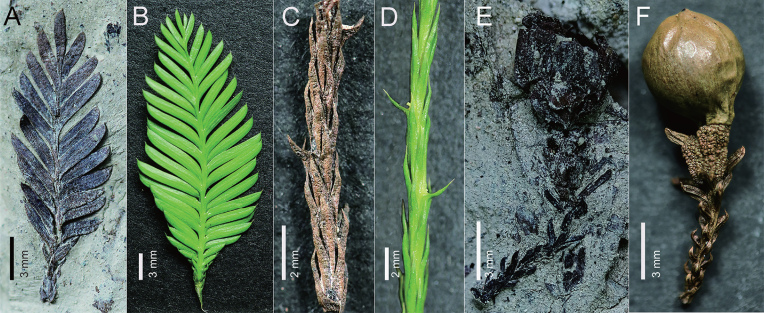
Different organs of *Dacrycarpus* fossils from Miocene of South China (A, C, E) and modern species *Dacrycarpus imbricatus* (Blume) de Laubenfels (B, D, F). (A) Short shoot of fossil *Dacrycarpus*, GP185; (B) short shoot of modern *Dacrycarpus*; (C) long shoot of fossil *Dacrycarpus*, GP286; (D) long shoot of modern *Dacrycarpus*; (E) ovuliferous shoot of fossil *Dacrycarpus*, GP277 and (F) ovuliferous shoot of modern *Dacrycarpus*.

There are three islands of particular importance for understanding the modern distribution of *Dacrycarpus*. The New Guinea highlands emerged from below sea level and began to build topography at ∼12 Ma [7], providing a land-bridge for Australia and Sulawesi. New Zealand was detached from Gondwanaland at *∼*80 Ma and segregated from its nearest neighbor, Australia, at ∼50–60 Ma [8]. Although the land area reduced significantly during the Oligocene, the fossil record and molecular data indicate that the then New Zealand was not completely submerged [9]. Reconstruction of early to middle Miocene in New Zealand suggests a subtropical to warm-temperate climate with higher terrestrial temperatures and rainfall than those of the present [10]. New Caledonia was insulated from the northeastern margin of Australia at ∼80 Ma, arriving its modern location at 50 Ma, and emerged ∼37 Ma after a long Paleocene and Eocene submergence [11]. Moreover, New Zealand was probably connected to New Caledonia by island chains or the now submerged Norfolk Ridge. The similar terranes between New Zealand and New Caledonia showed post-Permian structural continuity along the Norfolk Ridge [6]. Furthermore, the late Oligocene volcanic rocks from southern Norfolk imply that subduction-related volcanism occurred there at least 26 million years ago. Such a chain of islands would have facilitated immigration of *Dacrycarpus* between New Caledonia and New Zealand. The orogenic movement and island chain formation caused by plate collision provided a route for seed dispersal by endozoochory and ornithochory that would have allowed numerous taxa and not just *Dacrycarpus* to travel long distances via island chains.

These geological events are mainly based on the reconstruction of the paleogeography of Southeast Asia [6] and show that island chains may have connected Australia with Asia throughout the mid- to late Miocene, providing two main migration paths: (i) Australia–Lesser Sunda Islands–Java-Sumatra–Indochina; (ii) Australia–New Guinea–Sulawesi–Borneo–Sumatra–Indochina (Fig. 1).

Global climatic events that are associated with tectonism provide a dynamic context within which to view the evolution of plants. The Australia–Asia collision closed the wide and deep marine gap between the Sunda and Sahul shelves [6] and disrupted the Indonesian throughflow, so that moisture that previously moved between the Pacific Warm Pool and the Indian Ocean fell on Sundaland. The climate change from seasonally dry to ever-wet provided a chance for the development of the modern Malaysian flora, and for biotic exchange between Australia and Asia. The Middle Miocene Climatic Optimum provided a warmer climate, which extended habitats vertically and horizontally through mountain rainforests.

Physiological characteristics limit the range of plant habitats and distribution. Extant species inevitably inherit an ecological capacity from their ancestral species, as do their recent nearest relatives. As one of the least drought-tolerant conifer genera, extant *Dacrycarpus* has strict requirements for relatively ever-wet climates for both vegetative and reproductive growth [12]. This genus grows commonly on volcanic or ultramafic soils, waterlogged, peaty and acidic soils within mountain forests [2] or on fresh alluvial, often flooded, lowlands of New Zealand. The physiological characteristics of tolerance to being waterlogged and living in extreme environments enable them to avoid competition with most plant species and provides favorable conditions for their arrival and spread. Moreover, its edible, brightly colored and succulent podocarpium attracts the attention of birds and makes it a prime candidate for bird-assisted long-distance dispersal [3].

## DISPERSAL SCENARIO OF THE MODERN DISTRIBUTION OF *DACRYCARPUS*

*Dacrycarpus* may have originated in terminal Gondwana during the Late Cretaceous and became widespread in the region of Australasia–Antarctica–Patagonia during the Eocene [4,5]. Based on the available fossil records and modern distribution, we proposed a hypothetical scenario of the biogeographical history of *Dacrycarpus*. With the further break-up of Gondwana and global climate warming followed by pronounced cooling, the broad former distribution began to shrink and move northward. *Dacrycarpus* survived in New Caledonia and/or New Zealand as a relatively relictual species.

Molecular data show that the two species endemic to those areas (*D. dacrydioides* and *D. vieillardii*) generally are the earliest diverging members in the phylogenetic tree of the genus supporting New Guinea, Malesia, New Caledonia and Australia as the major source areas for the Pacific biota [13], so *Dacrycarpus* probably spread eastward to Melanesia from New Guinea by island-hopping. Plate collisions provided two routes of diffusion to Asia: (i) *Dacrycarpus* moved from Northern Australia west to the Lesser Sunda Islands via a volcanic island chain and subsequently spread to Java, Sumatra, Indochina and then arrived in South China; (ii) *Dacrycarpus* dispersed from Northern Australia to New Guinea, Sulawesi, Borneo, Sumatra, Indochina and then arrived in South China during the Miocene (Fig. 1).

## FLORISTIC EXCHANGE BETWEEN ASIA AND AUSTRALIA

For the Australia–Asia collision, from the mid-Miocene onwards, comparatively favorable conditions made for increasingly easy biotic exchanges [14], but floristic exchange showed a strong asymmetry: the number of out-of-Australia groups was far lower than the number of immigrants into Australia. Evidence for a Neogene movement into Asia is well documented, often from the late Miocene or later, including the migrations of the Haloragaceae, Chrysophylloideae (Sapotaceae), *Helicia* (Proteaceae) and Asian members of the Loranthaceae [14,15]. However, plant dispersal in the opposite direction (from Asia to Australia) is recorded in many cases, such as *Alocasia* (Araceae), *Aglaia* (Meliaceae), *Begonia* (Begoniaceae), *Margaritopsis* (Rubiaceae), *Pseuduvaria* (Annonaceae) and *Livistona* (Arecaceae) [16].

In addition, previous studies have also shown that most elements of the modern East Asian flora originated during the early Miocene [1], sometimes attributed to a Miocene uplift of Tibet and a Neogene onset of Asian monsoon circulation [17]. However, most of these studies proposed spread directions of individual taxa based only on molecular data without the support of fossil evidence. Only Sniderman and Jordan had been discussed in the exchange of rainforest taxa between Asia and Australia based on both fossil and extant data [14]. Moreover, the majority of these fossil records are pollen grains, which are able to travel a long distance, whereas megafossils that are invariably *in situ* preserved close to their growth site are confined to Asia or Australia. Although Wang and Ran comprehensively reviewed the evolution and biogeography of gymnosperms and indicated a possible migration route from Australia to Asia for 10 genera (including *Dacrycarpus*), their hypothesis lacks solid fossil and molecular evidence [18]. Here we further review fossil records of 151 genera of the Tropic Asia to Tropic Australia distribution taxa (Type 5) categorized by Wu *et al.* [19]. The results show that only 27 genera of the Tropic Asia to Tropic Australia distribution taxa are supported by fossil records (Supplementary Data); most of these genera are only distributed in the Northern Hemisphere, including *Adenanthera*, *Breynia*, *Dillenia*, *Desmos*, *Ailanthus*, *Dysoxylum*, *Toona*, *Tetrastigma*, *Buchanania*, *Semecarpus*, *Saccopetalum*, *Lagerstroemia*, *Cudrania*, *Madhuca*, *Hydrilla*, *Hippocratea*, *Harpullia*, *Xanthophyllum*, *Caryota*, *Livistona* and *Nypa*. And only *Baeckea* fossils are found in the Southern Hemisphere. Among these genera, *Ailanthus*, *Cedrela*, *Dillenia*, *Desmos*, *Xanthophyllum* and *Dysoxylum* are most likely to be involved in the Miocene floristic exchange based on their modern distribution. About five genera are distributed in both hemispheres, namely *Cinnamomum*, *Dacrycarpus*, *Helicia*, *Rhodomyrtus* and *Endiandra*. Although *Cinnamomum* has abundant fossils in different continents, at the moment, it is not possible to infer the exact origin center and migration route. But other genera have clear spatio-temporal orientation. *Rhodomyrtus* (Eocene of England and middle Eocene-Oligocene of southwestern Australia) and *Endiandra* (Paleogene of London Clay and the Eocene of Australia) show a clear trend of diffusion from north to south. *Helicia* (Paleogene of New Zealand and Holocene of China) and *Dacrycarpus* are showing the opposite trend. Apparently, *Rhodomyrtus* and *Endiandra* appear to have migrated between Asia and Australia earlier than the early Miocene. Only the exact fossil records of *Helicia* and *Dacrycarpus* prove their involvement in the Miocene floristic exchange. The discovery of our new fossil in South China is not only the first megafossil of *Dacrycarpus* in the Northern Hemisphere, but also provides direct evidence for long-distance dispersals and the floral exchange between Asia and Australia by the Miocene. Fossil and molecular data both show that *Dacrycarpus* is not the only taxa entering Asia or Australia by this course, suggesting that this event probably facilitated other bio-exchanges across the equator and contributed significantly to Neogene floral diversification in southern Asia, as shown in the *Dacrycarpus* pattern of the present study.

## SUMMARY

New *Dacrycarpus* megafossils from southern China provide solid evidence for long-distance dispersals from Australia to Asia during the Miocene. This trans-equatorial migration route long post-dates the India-Asia suture and seems to have resulted from the collision of the Australian Plate and Asian Plate after the late Oligocene. *Dacrycarpus* is unlikely to have been the only taxon entering Asia via this way, suggesting that this event probably facilitated other biotic exchanges across the equator and contributed significantly to Neogene plant diversity in southern Asia, namely the *Dacrycarpus* pattern.

## Supplementary Material

nwz060_Supplemental_File
