# Supplementary material for: Dacrycarpus pattern shedding new light on the early floristic exchange between Asia and Australia
Source: Natl Sci Rev. 2019 May 9;6(6):1086–90. doi: 10.1093/nsr/nwz060 (PMC8291558; doi:10.1093/nsr/nwz060)
Supplement: nwz060_Supplemental_File [file nwz060_supplemental_file.doc]

Supplementary data: Fossil records of 27 genera from the areal-type 5 (Tropic Asia to Tropic Australasia Oceania) of the Areal-types of the World families of seed plants (Wu et al., 2006)

| Family | Genus | Organ(s) | Age | Localities | Reference |
| --- | --- | --- | --- | --- | --- |
| Mimosaceae | *Adenanthera* | wood | Tertiary | Assam, India | [1] |
| Chamaesyce | *Breynia* | leaflet | Neogene | Nepal | [2] |
| Dilleniaceae | *Dillenia* | leaf | late Miocene | India | [3] |
|  |  | leaf | early Eocene | Belgium | [4]; [5] |
|  |  | leaf | middle to late Miocene | Nepal: Lower Siwalik beds of Koilabas | [6] |
|  |  | leaf | middle Miocene-Pliocene | west Bengal, India | [7] |
|  |  | leaf | Tertiary | Alaska | [8] |
| Annonaceae | *Desmos* | leaf | late Miocene | Yunnan, China | [9] |
| Annonaceae | *Saccopetalum* | leaf | Miocene | India | [10] |
|  |  | leaf | Oligocene | India | [11] |
| Simaroubaceae | *Ailanthus* | fruit | from Eocene to Pleistocene | Europe, North America, Asia | [12]; [13] |
| Meliaceae | *Dysoxylum* | flower with in situ pollen | early Miocene | New Zealand | [14] |
|  |  | leaflet | middle to late Miocene | India | [15] |
| Meliaceae | *Toona* | leaf, fruit and seed | from Eocene to Pliocene | Europe, Asia | [16]; [17]; [18] |
| Podocarpaceae | *Dacrycarpus* | leaflet, male and female cone | from Eocene to Pliocene | Australasia, China, South America | [19]; This paper |
| Vitaceae | *Tetrastigma* | megafossil | from Eocene to Pliocene | Europe and Japan | [20]; [21]; [22] |
| Anacardiaceae | *Buchanania* | leaf | late Miocene | western Nepal | [3] |
| Anacardiaceae | *Semecarpus* | leaf | late Oligocene | India | [23] |
| Lythraceae | *Lagerstroemia* | wood | Miocene | India | [24]; [25]; [26] |
| Moraceae | *Cudrania* | wood | late Pliocene | Shanxi, China | [27] |
| Sapotaceae | *Madhuca* | wood | mid Tertiary | India | [28] |
| Proteaceae | *Helicia* | fruit | Holocene | Nanjing, China | [29] |
|  |  | cuticle | early Paleocene | New Zealand | [30] |
| Hydrocharitaceae | *Hydrilla* | leaf | late Eocene | North Bohemian | [31]; [32] |
| Myrtaceae | *Baeckea* | leaf | Pleistocene | Tasmania | [33] |
| Myrtaceae | *Rhodomyrtus* | leaf | middle Eocene-Oligocene | southwestern Australia | [34] |
|  |  | leaf | Eocene | England | [35] |
| Celastraceae | *Loeseneriella/Hippocratea* | flower | from Miocene to 1000 ybp | The Dominican Republic | [36] |
| Sapindaceae | *Harpullia* | leaf | mid Miocene | Nepal | [3] |
| Polygalaceae | *Xanthophyllum* | wood | Miocene-Pliocene | India | [37] |
| Lauraceae | *Cinnamomum* | wood, leaf | from Late Cretaceous to Holocene | Europe, North America, Asia, Australasia | [38] |
| Lauraceae | *Endiandra* | fruit | Paleogene | London Clay | [16] |
|  |  | leaf | Eocene | Australia | [39] |
| Arecaceae | *Caryota* | seed | early Eocene | London clay | [16] |
| Arecaceae | *Livistona* | seed | Eocene | London Clay | [16] |
|  |  | seed | middle Eocene | Geiseltal, Germany | [16] |
|  |  | seed | early Miocene | Czech Republic Turów | [40] |
| Arecaceae | *Nypa* | fruit | Late Cretacous-Paleocene | Egypt | [41] |
|  |  | fruit | Eocene | Spain | [42] |

References

1. Prakash U, Tripathi PP. Fossil wood of *Adenanthera* and *Swintonia* from the Tertiary of Assam. *Curr Sci India* 1968; **37**: 115–116.

2. Awasthi N, Prasad M. Siwalik plant fossils from Surai Khola area, western Nepal. *Palaeobotanist* 1990; **38**: 298–318.

3. Gautam S. Studies on plant fossil from Siwalik Foreland Basin of Arjun Khola Area, Nepal and their plaeoclimatic and phytogeograpic emplication. *Ph.D. Thesis.* Allahabad University Department of Botany 2017.

4. Saporta GD, Marion AF. Révision de la flore heersienne de Gelinden. *Mém Cour Mém Sav Étrang Acad Sci Belg* 1878; **41**: 1–112.

5. Berry EW. The lower Eocene floras of southeastern North America. *US Geol Surv Prof Pap* 1916; **91**: 1–481.

6. Prasad M, Prakash U. Leaf impressions from the Lower Siwalik beds of Koilabas, Nepal. In: *Proceedings of the V^th^ Indian Geophytological Conference, Lucknow, 1983*. Special Publication 1984, pp. 246–256.

7. Antal JS, Awasthi N. Fossil flora from the Himalayan foothills of Darjeeling district, West Bengal and its palaeoecological and phytogeographical significance. *Palaeobotanist* 1993; **42**: 14–60.

8. Hollick A. The Tertiary floras of Alaska. *US Geol Surv Prof Pap* 1936; **182**: 1–185.

9. Tao JR, Zhou ZK and Liu YS. *The evolution of the Late Cretaceous – Cenozoic Floras in China.* Beijing: Science Press, 2000.

10. Prasad M. Siwalik (Middle Miocene) leaf impressions from the foothills of the Himalayas, India. *Tertiary Res* 1994; **15**: 53–90.

11. Awasthi N, Mehrotra RC. Oligocene flora from Makum Coalfield, Assam, India. *Palaeobotanist* 1995; **44**: 157–188.

12. Corbett S, Manchester S. Phytogeography and fossil history of *Ailanthus* (Simaroubaceae). *Int J Plant Sci* 2004; **165**: 671–690.

13. Su T, Jacques F and Ma HJ *et al*. **Fossil fruits of** Ailanthus confucii **from the Upper Miocene of Wenshan, Yunnan Province, southwestern China.** *Palaeoworld* 2013; **22**: 153–158.

14. Conran JG, Lee WG and Lee DE *et al*. Reproductive niche conservatism in the isolated New Zealand flora over 23 million years. *Biol Lett* 2014; **10**: 20140647.

15. Khan M, De B and Bera S. A fossil fern-leaflet of family Thelypteridaceae from the Middle Siwalik sediments of West Kameng district, Arunachal Pradesh. *J Bot Soc Beng* 2007; **61**: 65–69.

16. Reid EM, Chandler MEJ. *The London Clay Flora*. London: British Museum (Natural History), 1933.

17. Chandler MEJ. *The Lower Tertiary floras of southern England IV*. London: British Museum (Natural History), 1964*.*

18. Muellner AN, Pennington TD and Koecke AV *et al*. **Biogeography of** Cedrela **(Meliaceae, Sapindales) in Central and South America.** *Am J Bot* 2010; **97**: 511–518.

19. Wilf P. Rainforest conifers of Eocene Patagonia: attached cones and foliage of the extant Southeast Asian and Australasian genus *Dacrycarpus* (Podocarpaceae). *Am J Bot* 2012; **99**: 562–584.

20. Teodoridis V. Early Miocene carpological material from the Czech part of the Zittau Basin. *Acta Palaeobot* 2003; **43**: 9–49.

21. Chen I, Manchester SR. Seed morphology of modern and fossil Ampelocissus (Vitaceae) and implications for phytogeography. *Am J Bot* 2007; **94**: 1534–1553.

22. Habib S, Dang VC and Ickert-Bond SM *et al*. Robust phylogeny of *Tetrastigma* (Vitaceae) based on ten plastid DNA regions: Implications for infrageneric classification and seed character evolution. *Front Plant Sci* 2017; **8**: 590.

23. Srivastava G, Mehrotra RC. Oldest fossil of Semecarpus L.f. from the Makum Coalfield, Assam, India and comments on its origin. *Curr Sci India* 2012; **102**: 398–400.

24. Graham SA. **Fossil records in the Lythraceae.** *Bot Rev* 2013; **79**: 48–145.

25. Mehrotra RC, Srivastava G and Srikarni C. *Lagerstroemia* L. wood from the Kimin Formation (Upper Siwalik) of Arunachal Pradesh and its climatic and phytogeographic significance. *J Geol Soc India* 2015; **91**: 695–699.

26. Srivastava G, Gaur R and Mehrotra RC. *Lagerstroemia* L. from the middle Miocene Siwalik deposits, northern India: implication for Cenozoic range shifts of the genus and the family Lythraceae. *J Earth Syst Sci* 2015; **124**: 227–239.

27. Cao J, Cui H. [The Pliocene Flora from the Yushe Basin of Shanxi Province and its environmental significance](https://pdfs.semanticscholar.org/ee22/9fe3459e3de24527b40c8305d7dfe4cce86d.pdf). *Sci Geol Sinica* 1989; **9**: 369–375.

28. Prakash U, Tripathi PP. Fossil dicotyledonous woods from the Tertiary of eastern India**.** *Palaeobotanist* 1975; **22**: 51–62.

29. Kong ZC, Du NQ and Zhang YJ *et al*. Discovery of *Helicia* fossil flora and sporopollen assemblage of Baohuashan in Jurong County and its climatic and botanic significance (in Chinese). *Quatern Sin* 1991; **4**: 326–337.

30. Pole MS. The Proteaceae record in New Zealand. *Aust Syst Bot* 1998; **11**: 343-372.

31. Mai DH, Walther H. Die obereozänen Floren des Weisselster-Beckens und seiner Randgebiete. *Abh Staat Mus Miner Geol* *Dresden* 1985; **33**: 1–260.

32. Bowes G, Rao S and Estavillo G *et al*. C4 mechanisms in aquatic angiosperms: 22 comparisons with terrestrial C4 systems. *Funct Plant Biol* 2002; **29**: 379–392.

33. Jordan GJ, Barnes R and Hill RS *et al*. An Early to Middle Pleistocene flora of subalpine affinities in lowland western Tasmania. *Aust J Bot* 1993; **43**: 231–242.

34. Hill RS, Merrifield HE. An early Tertiary macroflora from West Dale, southwestern Australia. *Alcheringa* 1993; **17**: 285–326.

35. Bandulska H. On the cuticles of some recent and fossil Myrtaceae. *Bot J Linn Soc* 1931; **48**: 657–671.

36. Poinar GO, Chambers KL and Brown AE. *Hippocratea volubilis* (Celastraceae) in Cotui copal from the Dominican Republic. *J Bot Res Inst Texas* 2013; **7**: 375–379.

37. Awasthi N. Fossil wood of *Xanthophyllum* from the Cuddalore Sandstone near Pondicherry. *Palaeobotanist* 1986; **35**: 314–417.

38. Huang JF, Li L and van der Werff H *et al*. Origins and evolution of cinnamon and camphor: a phylogenetic and historical biogeographical analysis of the *Cinnamomum* group (Lauraceae). *Mol Phyl* 2016; **96**:33–44.

39. Keefe RL. The Brandy Creek fossil flora. *Ph.D. Thesis*. Victoria University 2012.

40. Czeczott H, Juchniewicz K. Flora kopalna Turowa koło Bogatyni II (4). Monocotyledonae Palmae. *Prace Muz. Ziemi, Prace Paleobot* 1975; **24**: 57–64.

41. El-Soughier MI, Mehrotra RC and Zhou ZY *et al*. *Nypa* fruits and seeds from the Maastrichtian–Danian sediments of Bir Abu Minqar, South Western Desert, Egypt. *Palaeoworld* 2011; **20**: 75–83.

42. Moreno-Dominguez R, Cascales-Miñana B and Ferrer J *et al*. First record of the mangrove palm *Nypa* from the northeastern Ebro Basin, Spain: with taphonomic criteria to evaluate the drifting duration. *Geol Acta* 2016; **14**: 101–111.
